# Partial hepatectomy in rats results in immediate down-regulation of p27Kip1 in residual liver tissue by transcriptional and post-translational processes

**DOI:** 10.3389/fphys.2013.00139

**Published:** 2013-06-13

**Authors:** Anne-Katrin Rohlfing, Karoline Trescher, Juliane Hähnel, Christian Müller, Jan-Peter Hildebrandt

**Affiliations:** Biotechnikum, Animal Physiology and Biochemistry, Ernst Moritz Arndt-University GreifswaldGreifswald, Germany

**Keywords:** cell cycle regulator, cyclin-dependent kinase inhibitor, Kip1, compensatory growth, liver regeneration, rat hepatocytes, cell proliferation

## Abstract

**Purpose:** The cyclin-dependent kinase (Cdk) inhibitor p27Kip1 may be involved in regulating re-entry of residual hepatocytes into the cell cycle upon loss of liver tissue by partial hepatectomy (PH). As yet, changes in Kip1 expression during the initial period following PH are not well-characterized. We investigated immediate changes in Kip1 mRNA and protein levels as well as changes in Kip1 phosphorylation in liver tissue within the relevant time window between surgery and the onset of DNA synthesis at 10–12 h.

**Methods:** We used real-time PCR, quantitative Western blotting, and immune histochemistry on tissue samples of adult rats obtained during or between 2 and 10 h after surgical removal of two thirds of the liver to analyze Kip1 mRNA or protein levels, respectively, or to quantify nuclear expression of Kip1.

**Results:** Kip1 mRNA was down-regulated within 4 h after PH by 60% and remained unchanged thereafter up to 10 h. With a lag phase of 2–3 h, Kip1-protein was down-regulated to a level of 40% of the control. The level of Thr187-phosphorylated Kip1 started to increase at 4 h and reached a maximum level at 8–10 h after PH. Kip1 immunoreactivity was observed in 30% of the hepatocytes before PH. Within 6–8 h after PH, more than half of the hepatocytes lost nuclear Kip1 signals. Kip1-specific micro-RNAs (miRNA221, miRNA222) were not changed upon PH.

**Conclusions:** A portion of hepatocytes in adult rats constitutively express Kip1 and down-regulate Kip1 immediately upon PH. This response involves transcriptional processes (loss of Kip1 mRNA) as well as accelerated degradation of existing protein (increase in pThr187-phosphorylation mediating polyubiquitinylation and proteasomal degradation of Kip1). Kip1 down-regulation occurs precisely within the intervall between surgery and onset of DNA synthesis which supports the hypothesis that it mediates activation of G0/0S-phase Cdk/cyclin-complexes and re-entry of hepatocytes into the cell cycle.

## Introduction

Loss of liver tissue in vertebrates is followed by the re-entry of the remaining hepatocytes into the cell cycle, DNA-replication, and cell division, a process termed compensatory hyperplasia (Fausto et al., 2006), which eventually results in restoration of the original liver mass. This process has been extensively studied in rodents which underwent surgical removal of approximately two thirds of the original liver mass (partial hepatectomy, PH; Higgins and Anderson, 1931). Extracellular signals, especially those mediated by the cytokines TNF-α, IL-1, or IL-6 (Jia, 2011), hepatocyte growth factor (Ping et al., 2006), or amphiregulin (Berasain et al., 2005), are required for priming residual liver cells to induce dedifferentiation and sensitization toward mitogens (Taub, 2004; Fausto et al., 2006; Michalopoulos, 2007). Mitogenic signals as well as removal of anti-mitogenic signals initiate cell cycle progression in partially dedifferentiated hepatocytes by altering expression of genes encoding transcription factors as STAT-3, AP-1, NFkB, forkhead box proteins, or the zinc finger transcriptional repressor Snail (Costa et al., 2003; Sekiya and Suzuki, 2011).

As in other proliferation-competent epithelial cells, the cyclin-dependent kinase (Cdk) inhibitor p27Kip1 (Kip1) is expressed in the nuclei of quiescent hepatocytes (Nakayama and Nakayama, 1998). Its down-regulation seems to be a prerequisite for the onset of DNA synthesis in residual liver cells upon PH (Michalopoulos and DeFrances, 1997; Hayashi et al., 2003). In general, mitogenic stimulation of epithelial cells results in phosphorylation of Kip1 at threonine residues, especially Thr187 (Sheaff et al., 1997; Fujita et al., 2003), which facilitates Kip1-polyubiquitinylation by the E3-ligases Skp2 (Xu et al., 2003; Kossatz et al., 2004), Ro52 (Sabile et al., 2006), or Kip-promoting complex (KPC; Kamura et al., 2004; Kotoshiba et al., 2005), respectively, and accelerates its proteasomal degradation (Carrano et al., 1999; Shirane et al., 1999). There are conflicting reports in the literature whether protein phosphorylation of Kip1 at Ser10 is instrumental in stabilization and nuclear retention of Kip1 (Deng et al., 2004; Kotake et al., 2005) or serves as a signal for nuclear export, cytosolic polyubiquitinylation, and proteasomal degradation (Lee and Kay, 2007; Lu and Hunter, 2010).

With respect to Kip1, there are conflicting reports in the literature about the time course of changes in protein abundance upon PH in rats or mice. Especially Kip1 expression during the initial phase of induction of compensatory growth (up to 12 h after PH) is not well-studied. Ehrenfried et al. (1997) did not find any changes in Kip1 during a period of 21 days after PH in rats. Cho et al. (1996) measured Kip1-abundance in residual rat liver tissue starting from 3 h after PH and reported an increase in the Kip1-protein level between 9 and 24 h. Similar results, an increase in Kip1 at 5 and 13 h after PH in rats or between 12 and 120 h after PH in mice, were reported by Pujol et al. (2000) or by Albrecht et al. (1998), respectively. In contrast, Alisi et al. (2003) found that Kip1 was down-regulated in a sustained fashion between 18 h (onset of S-phase) and 34 h (M-phase of the cell cycle) following PH. If down-regulation of Kip1 were indeed a relevant prerequisite for the onset of DNA synthesis and cell proliferation in the regenerating liver upon PH, changes in Kip1 abundance should occur early after PH between “priming” of the hepatocytes at 2–4 h after PH and the onset of DNA synthesis which starts ~12 h after PH (Gerlach et al., 1997; Pahlavan et al., 2006; Michalopoulos, 2007).

Since the expression of Kip1 has not yet been studied in detail during this time interval, we set out to study the immediate changes in Kip1 abundance in residual liver tissue after PH. We analyzed time courses of Kip1 mRNA by quantitative real-time PCR (qPCR) and of Kip1 protein as well as of Thr187- or Ser10-phosphorylated Kip1 by quantitative Western blotting. Immune histochemistry was used to assess the relative number of Kip1-positive nuclei in liver tissue and the PH-induced changes in nuclear Kip1expression levels and to relate these data to the expression of proliferating cell nuclear antigen (PCNA) as a marker for proliferating hepatocytes (Theocharis et al., 1994).

## Materials and methods

### Animals, partial hepatectomy

Rats [*Rattus norwegicus*, LEW.1W (Klöting, 1987)] were obtained from the animal facility of the University of Greifswald and kept with food and drinking water *ad libitum*. Rats were used at an age of 15–20 weeks. Animals were deprived of food for 24 h before the experiments and anaesthetized using diethyl ether. The procedure for removal of the median lobe and the left lateral lobe of the liver was performed as described (Higgins and Anderson, 1931) and took no more than 15 min. Operated animals were allowed to recover in darkened cages and were offered 20% glucose in their drinking water. During a second operation, the remaining liver tissue was removed and the animals were sacrificed. All tissue samples were briefly stored on ice, weighed, and immediately processed.

### RNA-preparation, qPCR, dot blot

Tissue (100 mg) was transferred into a tube containing 1 ml Trizol-reagent (Invitrogen, Karlsruhe, Germany), minced, and homogenized on ice for 20 s using an Ultraturrax T8 (IKA, Staufen, Germany) at 20,000 rpm. The extraction of total RNA was performed as described previously (Chomczynski and Sacchi, 1987). The RNA was suspended in diethyl pyrocarbonate-treated double-distilled water and stored at −20°C after determining the RNA-concentration and purity by gel analysis, spectrophotometry (BioPhotometer, Eppendorf, Hamburg, Germany) or, on randomly selected samples used for the analyses of microRNAs, using the “eukaryote total RNA nano”-assay (Agilent Technologies, Böblingen, Germany).

Equal amounts of total RNA (2 μg or 10 ng, respectively) were transcribed into cDNA using the “high capacity cDNA reverse transcriptase”-kit or the “TaqMan microRNA reverse transcription”-kit (Applied Biosystems, Darmstadt, Germany) according to the manufacturer's instructions. The resulting cDNA was used as the template for qPCRs using the ABI PRISM 7000 or 7500 Fast sequence detection system and pre-synthetized TaqMan gene expression assays for the rat Kip1 gene (Applied Biosystems 4351372), the human miRNA-221 (Applied Biosystems 4427975, assay-ID 000524), or the human miRNA-222 (Applied Biosystems 4427975, assay-ID 002276). TaqMan gene expression assays for rat beta-actin (Applied Biosystems 4352340E), 4.5S rRNA (Applied Biosystems 4427975, assay-ID 001716), and U6 snRNA (Applied Biosystems 4427975, assay-ID 001973) were used as the endogenous controls. Reagents and disposables for these assays were obtained from Applied Biosystems. For each experimental sample, the difference between the crossing threshold cycle value (Ct-value) of the qPCR-reaction containing the target-TaqMan probe and the internal control-probe was calculated (ΔCt) and subtracted from the respective value of the control sample obtained during the initial operation of the same rat (ΔΔCt).

To check whether expression of β-actin mRNA remained unaffected during the time course of liver regeneration after PH, dot blot experiments were performed. 5 μg of total RNA in equal volumes of sterile double-distilled water of all control and experimental samples were spotted onto nitrocellulose membrane, air-dried for 10 min and crosslinked to the membrane by exposure to UV-light for 3 min on a transilluminator (NU-72, Benda, Wiesloch, Germany). A DIG-labeled β-actin probe was generated using two oligonucleotide primers derived from the rat β-actin cDNA sequence (NM_031144, nucl. 452–470 or 1079–1094, respectively) and the DIG-DNA Labeling and Detection Kit (Roche Applied Science, Mannheim, Germany) according to the manufacturer's recommendations. Pre-hybridization (for 1 h) and hybridization (over night) were performed in hybridization bottles in a standard rolling hybridization oven (GFL 7601, GFL, Burgwedel, Germany) at 42°C. After hybridization, membranes were washed twice under low (5 min, room temperature, 300 mmol/l sodium chloride, 50 mmol/l sodium citrate, 0.1% w/v sodium dodecyl sulfate) or high (15 min, 70°C, 75 mmol/l sodium chloride, 12.5 mmol/l sodium citrate, 0.1% w/v sodium dodecyl sulfate) stringency conditions, respectively. The detection of hybridization signals was performed using the DIG Luminescent Detection Kit (Roche Applied Science, Mannheim Germany) according to the manufacturer's protocol. Membranes were exposed to Hyperfilm (GE Healthcare, Freiburg, Germany) and signals were scanned and densitometrically analyzed using 1D Phoretix software (Non-Linear Dynamics, Newcastle upon Tyne, UK). For control of even loading, membranes were stained with methylene blue solution (0.04% methylene blue, 500 mmol/l sodium acetate, pH 5.2) for 5 min and air dried. Methylene blue-signals were scanned and densitometrically analyzed.

### Protein preparation, western blotting

Tissue samples were minced on ice and homogenized under liquid nitrogen. The tissue powder was processed and soluble proteins were separated by SDS-gel electrophoresis as described previously (Hildebrandt et al., 1998).

Monoclonal p27Kip1 antibody (1 : 2500, BD Transduction Laboratories, Heidelberg, Germany), polyclonal p-Thr187Kip1 antibody or p-Ser10Kip1 antibody (1 : 5000, Santa Cruz Biotechnology, Santa Cruz, CA, USA) or monoclonal β-actin antibody (1 : 10,000, Sigma, Taufkirchen, Germany) were used as primary antibodies. Horseradish peroxidase-coupled sheep anti-mouse (or anti-rabbit-) antibody (1 : 6000, Amersham Bioscience, Freiburg, Germany) was used as the secondary antibody. Enhanced luminescence reagents (Perbio, Bonn, Germany) and Hyperfilm (GE Healthcare, Freiburg, Germany) were used to visualize antibody binding. Re-blotting of stripped nitrocellulose membranes with monoclonal β-actin antibody was performed to control for even loading of the gel lanes and as a tool to normalize the Kip1 signals on different blots. Signals on autoradiography films were scanned and densitometrically analyzed using 1D Phoretix software (Non-Linear Dynamics, Newcastle upon Tyne, UK).

### Immune histochemistry

Tissue samples were obtained as described above and fixed for 24 h at 4°C in a freshly prepared Bouin-solution containing 0.85% (w/v) picric acid, 9.5% (w/v) formaldehyde and 0.05% (v/v) acetic acid followed by removal of excess picric acid by repeated washing with ethanol (70%, v/v). Dehydration and paraffin embedding were performed using an automated tissue processor (Histokinette, Reichert & Jung, Germany). Sections (5 μm) of control tissue and those of tissue samples taken at different times after PH were mounted pairwise on the same slide, so that the following processing steps were exactly the same for experimental and control samples. Tissue sections were processed as described previously (Hildebrandt et al., 1998; Rohlfing et al., 2005) using p27Kip1-specific monoclonal antibodies (1: 200, BD Transduction Laboratories, Heidelberg, Germany) or PCNA-specific monoclonal antibodies (1: 500, Sigma-Aldrich, Munich, Germany) and viewed using a Nikon Eclipse TE300 microscope and a Nikon DXM1200 digital camera.

For semi-quantitative analyses of Kip1 expression, all parameters as described below were determined in standard size images at six randomly selected, non-overlapping positions in each tissue section. Total numbers of nuclei counted in each tissue section were between 160 and 250. The respective mean represented one data point in the final calculations. Nuclei that were not Kip1-positive could easily be detected without counterstaining since the nuclear plasma appeared opaque and was different in structure than the hepatocyte cytosol which appears slightly granulated. The number of cells with Kip1-positive nuclei (threshold: density higher than cytosolic density) was counted and expressed as a percentage of the total number of nuclei within one image. The intensities of Kip1-related signals in nuclei and cytosol of randomly selected cells (6 per image) were determined in representative areas of 100 pixels using the density function of a commercial graphics program (Corel Photo Paint). Since we did not observe any systematic changes in cytosolic Kip1 abundance in control or PH samples, we subtracted means of cytosolic signal densities from the means of nuclear staining intensities to compensate for potential differences in staining intensity.

### Statistics

Four rats per time point were operated and their tissue samples (Con, PH) were analyzed pair-wise. Data are expressed as means ± SD (standard deviation). ANOVA was used to test means at different times for significant differences from each other. Individual means were tested for significant differences using Student's *t*-test or the Mann-Whitney U-test when the *F*-test indicated that variances of samples were different. Means were considered to be significantly different at *p* < 0.05.

## Results

**Figure 1:**
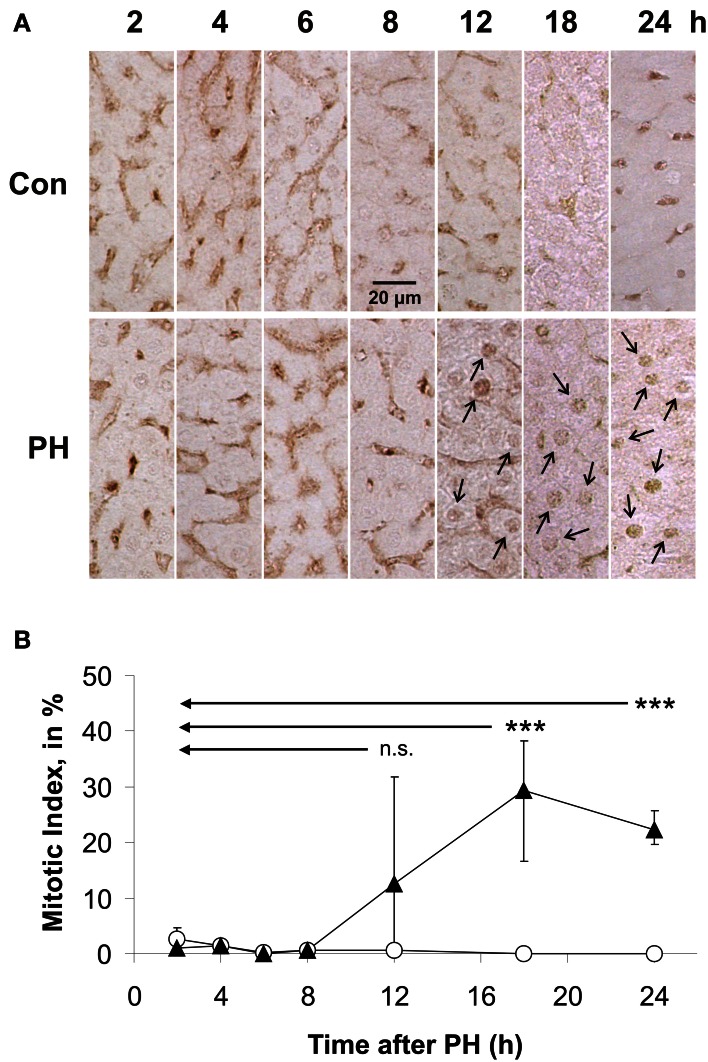
**Immune histochemical detection and quantification of proliferating cell nuclear antigen (PCNA) in paraffin sections of rat liver obtained during or at different times after partial hepatectomy**. PCNA-positive cells were counted in images of PCNA-antibody labeled liver sections. Data were obtained from images of control tissue prepared during initial surgery and from images of experimental tissue samples obtained during the second operation at different times after PH. **(A)** Representative example images of control tissue (Con) and residual liver tissue at 2, 4, 6, 8, 12, 18, or 24 h after partial hepatectomy (PH), respectively, are shown. Examples of PCNA-positive nuclei are labeled by arrows. **(B)** Number of hepatocytes showing PCNA-positive nuclei normalized to the total number of cells in the image in % (mitotic index). Data of the Con-time series are labeled with open circles, those of the hepatectomized animals (PH) with triangles. Means and standard deviations were calculated from results of tissue preparations from *n* = 4 different animals (6 counting replicates for each *n*) (n.s., *p* ≥ 0.05; ^***^p < 0.001).

Immune histochemical analyses using antibodies directed against PCNA (Figure 1) revealed that PCNA was not at all expressed in rat liver tissue obtained during the initial operation (Con). In residual liver tissue of PH rats, the onset of nuclear PCNA expression occurred ~12 h after PH (Figure 1; PCNA-positive nuclei are marked with arrows) indicating that labeled cells were in S-phase of the cell cycle. This finding defined the time window in which changes in Kip1 protein abundance that may be relevant for lifting the Kip1-mediated inhibition of Cdks at the G0/S-checkpoint occurred. Therefore, we focussed on the time intervall between the initial surgery and 10 h after PH in our further work.

**Figure 2:**
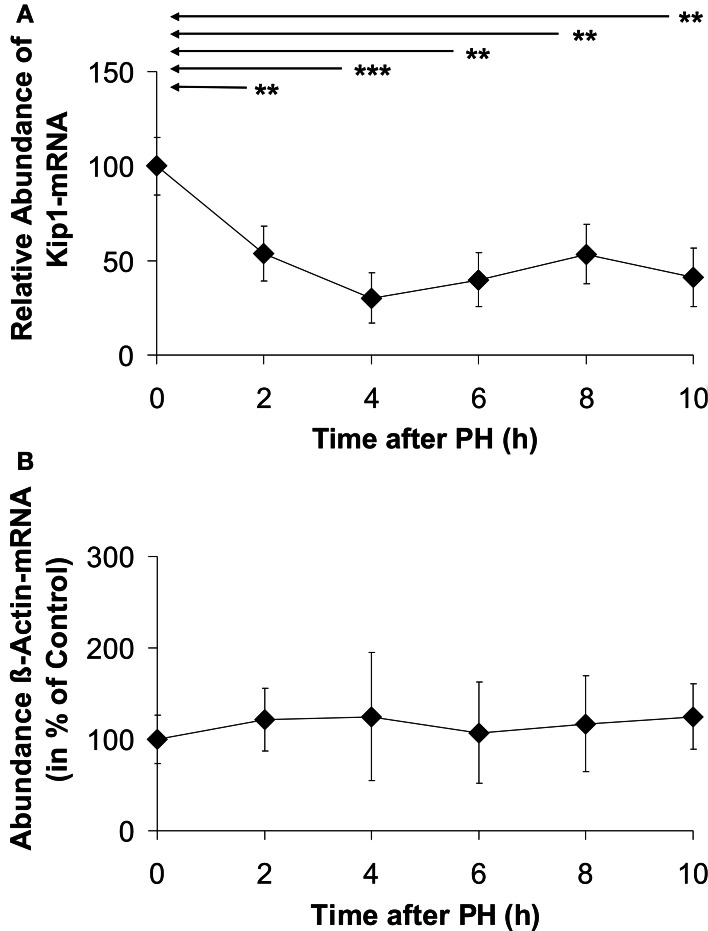
**Abundance of Kip1- (A) or β-actin (B) mRNAs in residual liver tissue at different times after partial hepatectomy. (A)** Data represent differences of the ΔCt-values (ΔΔCt-method) of Kip1- and β-actin-specific qPCR-signals. **(B)** Data represent densitometric signals obtained from dot blots using equal amounts of total RNA extracted from the tissue samples hybridized to a digoxigenin-labeled β-actin-specific probe. Data are expressed as percentages of the control values at 0 h. The data points represent means ± SD of *n* = 4 different preparations of tissue, each analyzed at least in triplicate (^**^p < 0.01; ^***^p < 0.001). No significant differences in β-actin mRNA abundance were detected in residual liver tissue of hepatectomized animals **(B)**.

There was a steep and statistically significant decline in Kip1 mRNA abundance to 55% of the initial level within 2 h after PH (Figure 2). The lowest level of Kip1 mRNA of ~40% of the control was reached at 4 h after PH and was maintained up to 10 h. Densitometric quantification of dot blots using the β-actin probe did not reveal any significant differences between β-actin mRNA abundances in control- and in any of the PH-samples (Figure 2B) indicating that β-actin mRNA was a suitable endogenous reference for the quantification of Kip1 mRNA in the qPCR experiments (Figure 2A). Confirming this conclusion, we found that the ΔCt-values obtained in the qPCR experiments using the β-actin specific probes did not show any differences between control samples obtained during the initial operation and the samples obtained at different times after PH (results not shown).

**Figure 3:**
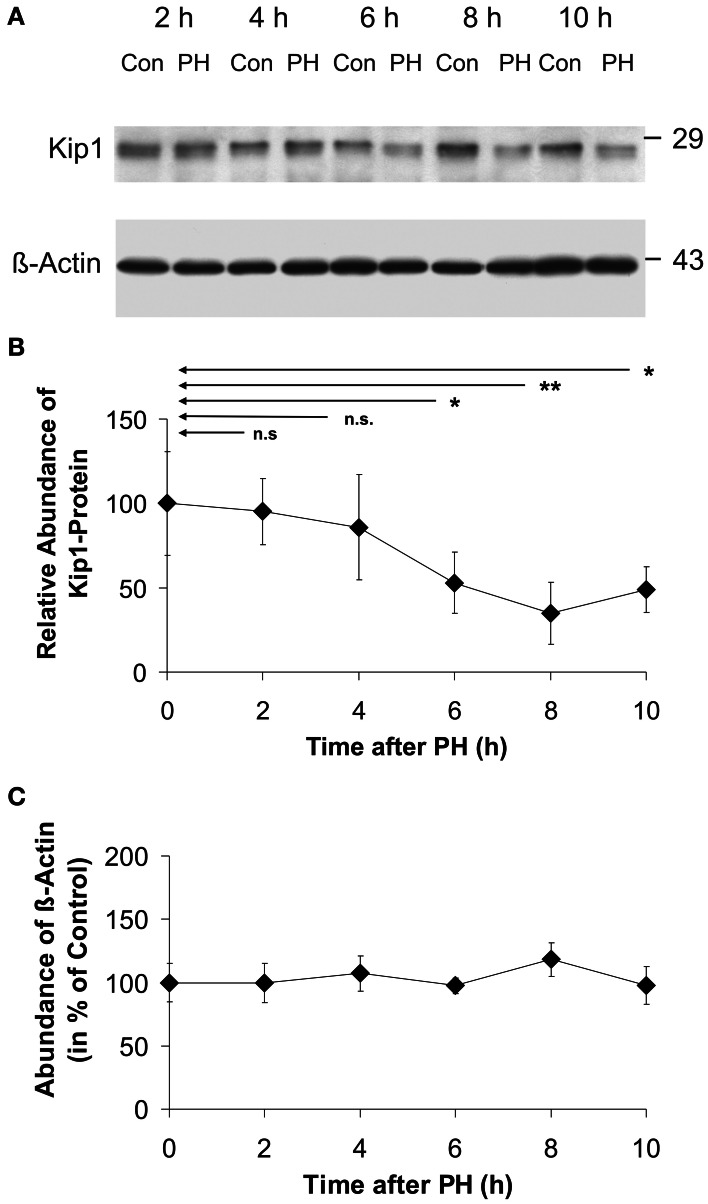
**Abundance of p27Kip1 or β-actin in residual liver tissue at different times after partial hepatectomy. (A)** Representative Western blots (using p27Kip1-specific or β-actin-specific monoclonal antibodies) of protein extracts from rat liver control samples (Con: tissue obtained during initial surgery) and in residual liver tissue at different times after PH (PH). The marks on the right show the positions of the protein standards with molecular masses as indicated. **(B)** Densitometric signals obtained from Western blots using equal amounts of protein extracted from the tissue samples as described in the legend to A and p27Kip1-specific monoclonal antibodies, normalized to the respective β-actin signals. **(C)** Densitometric signals obtained from Western blots using β-actin-specific monoclonal antibodies. Data in **(B)** and **(C)** are expressed as percentages of the control values at 0 h. The data points represent means ± SD of *n* = 4 different preparations of tissue, each analyzed at least in triplicate (n.s., *p* ≥ 0.05; ^*^p < 0.05; ^**^p < 0.01). No significant differences in β-actin abundance were detected in residual liver tissue of hepatectomized animals **(C)**.

As shown in the representative Western blot image (Figure 3A) and in the time course of Kip1-protein expression (Figure 3B), there was a statistically significant decline in Kip1 protein to ~50% of the initial level at 6 h after PH. The low level was maintained until 10 h after PH. Densitometric quantification of β-actin in Western blots (Figure 3C) did not reveal any significant differences between β-actin abundances in control- and in any of the PH-samples indicating that β-actin was a suitable endogenous reference for the quantification of Kip1 in quantitative Western blot experiments.

**Figure 4:**
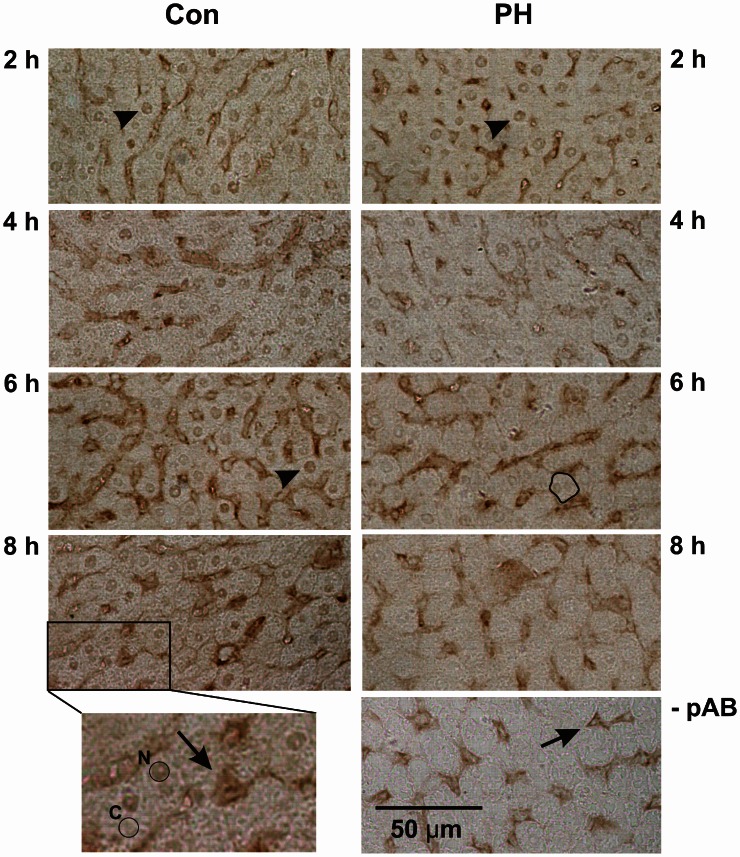
**Immune histochemical detection of p27Kip1 in paraffin sections of rat liver tissue obtained during or at different times after partial hepatectomy**. Representative example images of control tissue (Con, obtained during the initial operation) and residual liver tissue at 2, 4, 6, or 8 h after partial hepatectomy (PH), respectively, are shown. The image on the right at the bottom (-pAB) shows an example image developed without primary antibody which, in comparison with the control images on the left, shows lack of specific nuclear Kip1-staining, but retention of stain in the liver sinusoids (arrow) indicating that these signals are non-specific. Bottom left: enlarged area of the panel above: the encircled area labeled “N” marks a Kip1-positive hepatocyte nucleus, the encircled area labeled “C” marks a representative area of the cytosol of a neighboring cell. Densitometric analyses of such areas in all samples are reported in Figure 5. Non-specific staining (see above) occurred in the lining of the sinusoids (arrow). Specific labeling of nuclear Kip1 (examples marked by arrowheads) occurred in all samples taken from control animals (Con) and in tissue obtained from animals 2 h after PH, but not at later time points after PH (as an example, the outline of a hepatocyte containing a Kip1-negative nucleus is marked in the PH image at 6 h).

Immune histochemistry was used to determine the subcellular localization of Kip1 in hepatocytes during the initial phase of regenerative growth upon PH (Figure 4). Despite pre-incubation of tissue sections with hydrogen peroxide, there was still residual labeling of structures associated with the lining of sinusoids (Figure 4, insert, arrow), while only background staining was observed in the cytosolic regions of the liver cells (Figure 4, insert, encircled area labeled “C”). Control experiments in buffer solutions without Kip1-specific primary antibody showed that sinusoid-associated labeling was still present (Figure 4, -pAB, arrow) which indicates that it was non-specific. Use of an alternative secondary antibody (biotin-linked anti-mouse IgG #115-065-003 obtained from Dianova, Hamburg, Germany) showed the same pattern of non-specific labeling (results not shown). As the sinusoid-associated non-specific staining was not interfering with our analyses, we did not make further attempts to minimize it. The expression level of Kip1 was assessed by measuring the density of Kip1-specific signals in the nuclear region of individual liver cells (Figure 4, insert, encircled area labeled “N”). Liver tissue prepared during the initial operation was used as the control for each time point (Figure 4, Con) and compared with tissue of the same animal obtained during the second operation at different times after PH (Figure 4, PH). Direct comparison of Con with PH tissue samples revealed that Kip1-specific staining was present in both types of preparations at 2 h (examples of labeled nuclei are marked by arrowheads). Starting at 4 h, Kip1-positive nuclei in the PH tissue sections became sparse, an effect that became very obvious at 6 h (example of stained nucleus in control tissue marked by an arrowhead, outline of a single hepatocyte containing an unstained nucleus in the respective PH tissue sample). At 8 h upon PH, there were virtually no Kip1-positive nuclei visible in the PH tissue sections. In contrast, Kip1-positive nuclei were visible in all of the respective control samples (Con).

**Figure 5:**
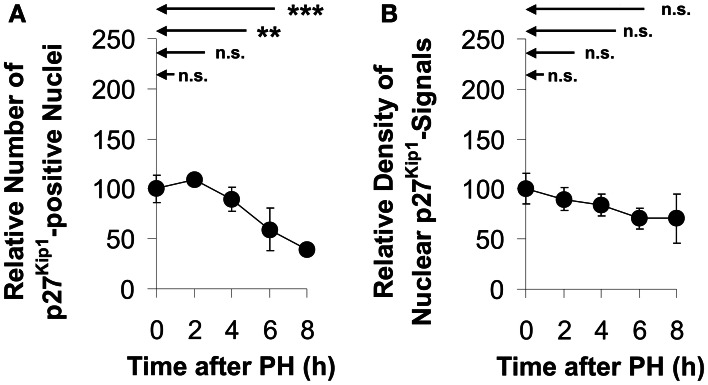
**Quantitation of Kip1-positive nuclei and nuclear Kip1 staining densities in sections of rat liver tissue obtained at different times after partial hepatectomy**. Kip1-positive nuclei were counted in and densitometric analyses were made on images of Kip1-antibody labeled liver sections. The Kip1-specific staining intensity in the nuclear region was determined as described in the legend to Figure 4. Data were obtained from images of control tissue prepared during initial surgery and from images of experimental tissue samples obtained during the second operation at different times after PH. **(A)** Number of hepatocytes showing Kip1-positive nuclei normalized to the total number of cells in the image. **(B)** Signal intensities of nuclear Kip1 in PH-samples relative to those in control tissue. Data are presented as percentages of the control values at 0 h. Means and standard deviations were calculated from results of tissue preparations from *n* = 4 different animals (6 counting replicates for each *n*) (n.s., *p* ≥ 0.05; ^**^p < 0.01; ^***^p < 0.001).

As shown in control sections prepared from tissue obtained during the initial operation (Con), approximately one third of all liver cells had low, but detectable levels of Kip1 in the nuclei (cf. encircled area labeled “N” in Figure 4, insert). The number of Kip-positive nuclei decreased to less than 50% of the initial values between 4 and 8 h after PH (Figures 4, 5A) indicating that the major portion of the residual liver cells down-regulated nuclear Kip1 upon PH, while those few hepatocyte nuclei which remained Kip1-positive did not substantially change expression levels of Kip1 within 8 h upon PH as indicated by densitometric analyses of nuclear Kip1-labeling (Figure 5B). The cytosolic space of liver cells (cf. encircled area labeled “C” in the insert of Figure 4) did not reveal any differences in Kip1-signal intensity between control and experimental samples after PH (data not shown).

**Figure 6:**
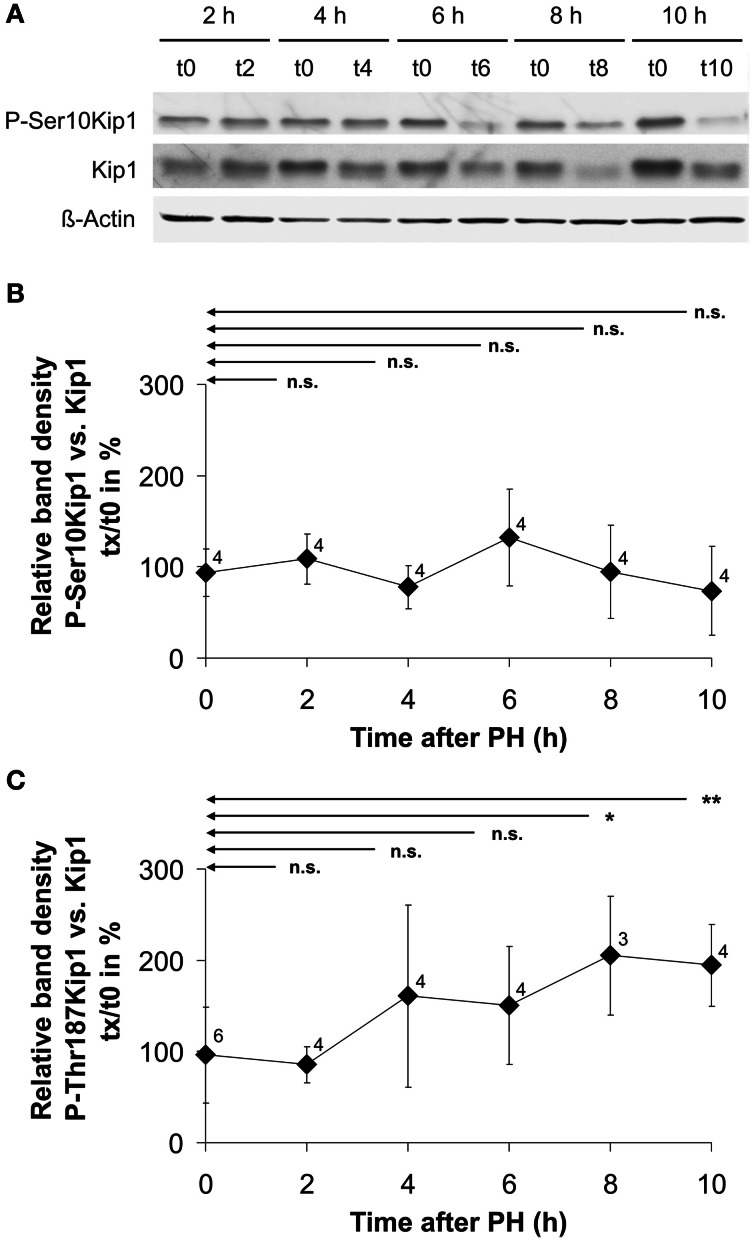
**Example image of a Western blot for the quantification of Kip1 phosphorylation level (A) and Kip1 phosphorylation levels at Ser10 (B) or Thr187 (C) in rat liver tissue obtained during PH (t0) as well as in residual liver tissue at different times after partial hepatectomy (tx). (A)** Proteins were extracted from samples of liver tissue obtained from individual rats during partial hepatectomy (t0) or during the second operation after different times of regeneration of residual liver tissue in the animals (tx). Blots were probed using phospho-specific Kip1 antibodies (P-Ser10Kip1) and, upon stripping, with antibodies against Kip1 (Kip1) and antibodies against β-actin (β-Actin). The latter signals were used as loading controls. For generating the diagrams **(B)** and **(C)**, the optical density of the P-Kip1 bands of each individual animal was normalized to that of the Kip1 bands (expressed in %). Plotted in the graphs are the percentages of P-Kip at the different times after PH relative to the amount of P-Kip under control conditions (0 h). Data points represent means ± SD of different tissue samples from n different animals, each analyzed at least in triplicate (n.s., *p* ≥ 0.05; ^*^p < 0.05; ^**^p < 0.01).

Since Kip1 phosphorylation at Ser10 or Thr187 has been implicated in nuclear export, polyubiquinylation, and degradation of Kip1, we measured the phosphorylation state of Kip1 during the initial 10 h following PH. Western blot experiments on extracts of residual liver tissue using phospho-Ser10-specific Kip1 antibodies revealed that Ser10 phosphorylation of Kip1 did not significantly change compared with the control levels (Figures 6A,B). Probing the same samples using phospho-Thr187-specific Kip1 antibodies (Figure 6C) we observed an increase in the phosphorylation level of Kip1 between 4 and 10 h after PH. Although the level of significance (compared with the controls at 0 h) was reached only for the samples at 8 or 10 h, respectively, this indicates that Thr187-phosphorylation was up-regulated just before the level of total Kip1 started to decline at 4–6 h after PH (Figure 3B).

**Figure 7:**
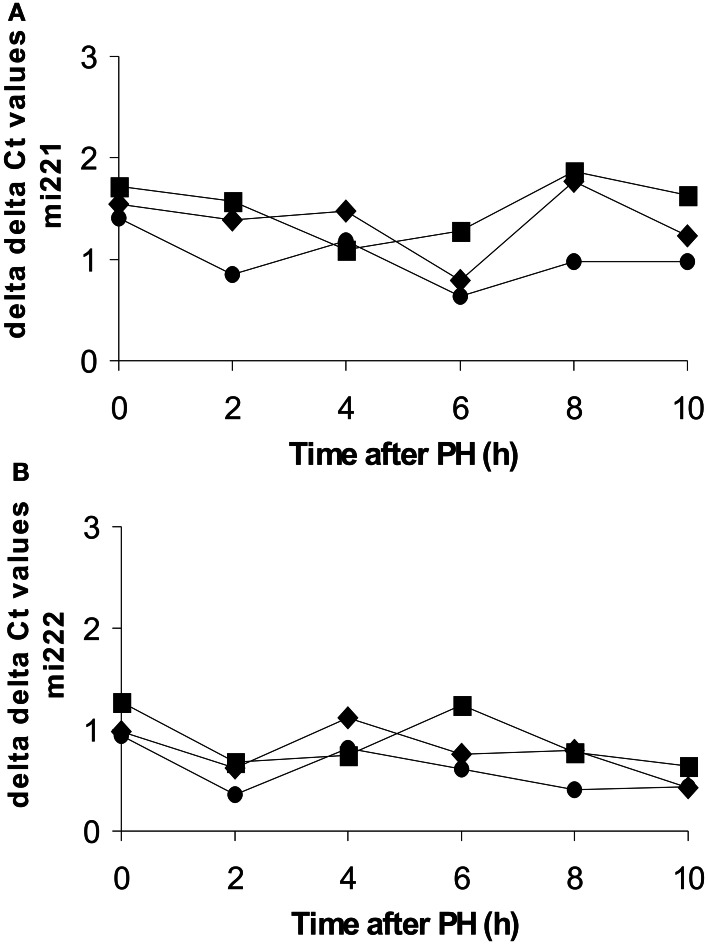
**Abundances of miRNA221 or miRNA222 in total RNA preparations from rat liver during or at different times after partial hepatectomy as determined by qPCR**. Data are expressed as ΔΔCt values for miRNA221 **(A)** or miRNA222 **(B)** using 4.5 S-RNA (diamonds), U6-snRNA (squares), or β-actin mRNA (full circles) as internal standards. Each data point represents the mean of the results of 2 replicates performed on a sample of total RNA obtained from one individual animal per time point.

To test the hypothesis that the rapid down-regulation of Kip1 mRNA may be mediated by opposite changes in Kip1-specific microRNAs (post-transcriptional mechanism), we quantified the abundances of mi221- and mi222-RNAs using TaqMan probes developed against the human RNAs in quantitative PCR assays. The stretches of Kip1-RNA sequence covered by the TaqMan probes are virtually identical or at least very similar in humans and rats (Iwanaga et al., 2006; Galardi et al., 2007; Kim et al., 2012). We measured microRNA abundances against three internal standards. As shown in Figure 7, ΔΔCt-values were distributed evenly around 1 for both types of microRNAs in the control samples obtained during the initial operation (0 h) and in all of the samples taken at different time points after PH (2–10 h). This indicates that liver cells did not up-regulate Kip1-specific microRNAs to down-regulate the expression of Kip1.

## Discussion

Kip1 is expressed in normal liver (Cho et al., 1996; Albrecht et al., 1998; Alisi et al., 2003) and functions as a block in the hepatocyte cell cycle by inhibition of Cdks at the G0/S-checkpoint (Hayashi et al., 2003). Upon PH, up to 60% of the remaining hepatocytes re-enter the cell cycle (Gerlach et al., 1997) to replace lost hepatocytes. Increases in DNA synthesis have been observed as early as 12 h after PH (Gerlach et al., 1997; Pahlavan et al., 2006). As we were able to reproduce these findings (Figure 1), we reasoned that signals driving residual hepatocytes into the cell cycle by down-regulation of Kip1 must occur between priming of the residual liver cells right after the initial surgery and the onset of DNA-synthesis at 12 h after PH. Therefore, we expected to detect a loss of Kip1 in the residual liver tissue within the period between the initial operation (0 h) and approximately 10–12 h after PH.

Our data obtained by qPCR (Figure 2), Western blotting (Figure 3), and immune histochemistry (Figure 4) indicate that the primary reason for Kip1 down-regulation may be a loss in mRNA, most likely by attenuation of transcription of the *kip1* gene followed by down-regulation of Kip1 protein ~2 or 3 h later. Recently, it became apparent that microRNAs, especially miRNA-221 and miRNA-222, may be important inhibitors of Kip1 translation (Galardi et al., 2007; Martínez-Sánchez and Gebauer, 2010). Thus, we performed qPCR analyses of these microRNAs in total RNA isolated from rat liver samples obtained at or at different times after PH. If such miRNAs would have had an impact on down-regulation of residual Kip1 mRNA-translation, we expected to find an up-regulation of one or both of these microRNAs during the time period between PH and 2 h after PH (before mRNA abundance actually declined, Figure 2A). We could, however, not find any obvious changes in miRNA-221 or miRNA-222 in residual liver tissue over the entire experimental period (Figure 7) indicating that these microRNAs do not contribute to the regulation of Kip1 abundance in residual liver tissue upon PH.

The observed loss in Kip1 protein may have resulted from the combination of lower translation rate as a consequence of the decline in mRNA abundance and accelerated degradation of existing Kip1. There was no accumulation of Kip1 in the cytoplasm of cells between 2 and 12 h after PH (Figure 4, insert, encircled area labeled “C”; results not shown) indicating that Kip1 is not just exported from the nucleus into the cytosol, but must have been removed from the cells altogether. It is, therefore, likely that PH induces progressive Kip1 ubiquitinylation and proteasomal degradation as previously shown for other vertebrate cell types (Shirane et al., 1999; Furstenthal et al., 2001; Rohlfing et al., 2005) and as described for mouse hepatocytes upon PH (Minamishima et al., 2002). Using phospho-specific Kip1 antibodies, we observed that Ser10 phosphorylation of Kip1 remained unchanged during the initial 8 h following PH (Figures 6A,B) indicating that Ser10 phosphorylation may not be involved in mediating changes in Kip1 abundance in residual liver cells upon PH. Thr187 phosphorylation of Kip1, however, was up-regulated starting at 4 h with a maximum at 8 h after PH (Figure 6C) indicating that Kip1 may indeed have undergone accelerated polyubiquitinylation and proteasomal degradation. Thus, it is likely that the rapid loss of Kip1 in residual liver cells following PH (Figures 3A,B) is the result of transcriptional (decline in mRNA abundance) and post-translational effects (acceleration of degradation of existing Kip1 protein).

In the quiescent liver, approximately one third of mature hepatocytes contain nuclei which stain positively for Kip1 (Figure 4). There was a decline in the number of cells with Kip1-positive nuclei in residual liver tissue between 4 and 8 h after PH (Figure 5A), but only moderate reduction in the Kip1-specific staining in nuclei that remained Kip1-positive (Figure 5B). This indicates that the reduction in total Kip1 protein in residual liver tissue at 6–10 h after PH (Figure 3B) was due to the removal of Kip1 from previously Kip1-positive nuclei. The few remaining hepatocytes with Kip1-positive nuclei, however, did not show major changes in nuclear Kip1 abundance. It may be these latter cells that maintain liver metabolism during the initial phases of compensatory growth.

A quantitative comparison of total Kip1 abundance in liver tissue (Figure 3) and in hepatocytes (Figure 4) indicates that the loss in total Kip1 may be fully explained by the loss of Kip1 from hepatocytes and that non-parenchymal cell types in the liver as endothelial-, duct-, Ito-, or Kupffer-cells, which have been shown to proliferate in response to PH (Theocharis et al., 1994; Malik et al., 2002; Baier et al., 2005; Duncan et al., 2009), do not significantly contribute to changes in Kip1 abundance.

Our immune histochemical data show that differentiated hepatocytes down-regulate Kip1 after PH (Figure 4). As a result, much of the compensatory growth in residual liver tissue may occur through cell proliferation of these parenchymal cells. This confirms the hypothesis (Alison et al., 2004) that subsets of hepatoblasts or hepatocytes which are permanently present in liver parenchyma are responsible for PH-mediated compensatory cell proliferation and that infiltration of hematopoietic or other types of stem cells (Duncan et al., 2009) into the residual liver tissue is not necessarily required for compensatory growth.

In summary, down-regulation of Kip1 in the hepatocytes by mechanisms involving transcriptional and post-translational processes occurs precisely within the intervall between surgical removal of liver tissue and the onset of DNA replication in preparation for compensatory cell proliferation which supports the hypothesis that loss of Kip1 mediates activation of G0/S-phase Cdk/cyclin-complexes and re-entry of hepatocytes into the cell cycle.

### Authorization for the use of experimental animals

The experiments were performed in accordance with German animal welfare laws. Permission for animal experimentation was granted by Landesamt für Landwirtschaft, Lebensmittelsicherheit und Fischerei Mecklenburg-Vorpommern, LALLF M-V/TSD/7221.3-1.1-047/05.

### Conflict of interest statement

The authors declare that the research was conducted in the absence of any commercial or financial relationships that could be construed as a potential conflict of interest.
